# Food allergy and anaphylaxis – 2053. New diagnostic algorithms that will reduce the need for oral food challenges

**DOI:** 10.1186/1939-4551-6-S1-P136

**Published:** 2013-04-23

**Authors:** Rachel Peters, Lyle Gurrin, Shyamali Dharmage, Mimi Tang, Jennifer Koplin, Margaret Danchin, Harriet Hiscock, Katie Allen

**Affiliations:** 1 Department of Paediatrics, University of Melbourne, Australia; 2 Centre for MEGA Epidemiology, University of Melbourne, Australia; 3 Murdoch Childrens Research Institute, Australia; 4 Centre for Community Child Health, Royal Children's Hospital, Australia; 5 Department of Allergy and Immunology, the Royal Children's Hospital, Parkville, Australia

## Background

Diagnosis of food allergy relies on clinical history, tests for allergen specific IgE and oral food challenges. A recent reaction consistent with IgE-mediated symptoms in conjunction with a positive allergen-specific IgE test (either skin prick test (SPT) or serum allergen specific IgE (sIgE)) is usually sufficient to diagnose food allergy. In the absence of a reaction history, a positive skin prick test or serum allergen-specific IgE test may not necessarily indicate clinical allergy and it is often necessary to perform resource-intensive and potentially dangerous oral food challenges. We aimed to develop diagnostic algorithms in infants, using sIgE testing, that will minimize the requirement for food challenges and resource utilization of over burdened allergy services.

## Methods

HealthNuts is a population-based, longitudinal food allergy study. Infants were recruited from council-run immunization sessions and underwent SPT to four allergens; those with any evidence of sensitisation were invited to undergo oral food challenges and sIgE testing. Diagnostic pathways of food allergy were identified through expert consultation. Predictive modeling was used to develop sIgE thresholds for each pathway that could accurately identify challenge-confirmed allergic and tolerant infants.

## Results

Three diagnostic pathways were identified: (A) history of immediate reaction consistent with IgE-mediated food allergy, (B) early-onset eczema in the absence of reaction history, and (C) family history of food allergy in the absence of reaction history. Clinical decision points for egg sIgE testing reduced the need for oral food challenges in pathway A by 89% (95% CI 82-95), pathway B by 82% (95% CI 75-87%) and pathway C by 65% (95% CI 52-76%). Clinical decision points for peanut sIgE testing reduced the need for oral food challenges in pathway A by 81% (95% CI 58-95%), pathway B by 47% (95% CI 39-55%) and pathway C by 71% (95% CI 58-92%). Infants who fell into the indeterminate category (sIgE result between the two thresholds), would still require assessment by specialist allergy services.

## Conclusions

Our new diagnostic algorithms were highly efficient in minimising oral food challenges and were reliable in a population-based setting.

